# An Explainable Transformer-Based Framework for Lung Cancer Classification and Automated Radiology Report Generation from Multi-Slice CT Images

**DOI:** 10.3390/biomedicines14051103

**Published:** 2026-05-13

**Authors:** Oguzhan Katar, Tulin Akbalik, Ozal Yildirim

**Affiliations:** 1 Graduate School of Natural and Applied Sciences, Firat University, Elazig 23119, Turkey; 2 Department of Software Engineering, Firat University, Elazig 23119, Turkey; ozalyildirim@firat.edu.tr; 3 Department of Radiology, Fethi Sekin City Hospital, Elazig 23119, Turkey

**Keywords:** vision transformer, explainability, multi-slice CT, radiology report generation, lung cancer

## Abstract

**Background/Objectives:** Lung cancer is one of the most common and lethal malignancies worldwide. Early detection remains challenging due to its variable biological behavior. Computed tomography (CT) is the primary imaging method used for early detection. However, the manual interpretation of CT scans is constrained by several challenges such as reliance on expert experience, increasing clinical workload, and considerable variability among observers. **Methods:** This study introduces an explainable transformer-based framework capable of distinguishing among the three principal clinical categories of lung cancer (small-cell lung cancer, non-small-cell lung cancer, and normal) while simultaneously generating automated radiology reports from CT images. In contrast to conventional single-slice methodologies, the proposed model employs a multi-slice volumetric encoding strategy that captures spatial continuity and anatomical relationships across the CT slices. Visual features extracted by a ViT-based encoder are transformed into a compact patient-level representation through a Learnable Query Attention Pooling (LQAP) mechanism, and this unified representation is subsequently used for both three-class prediction and report generation with a GPT-2-based decoder. To enhance explainability, slice-wise Grad-CAM maps are produced, visually highlighting the anatomical cues that guide the model’s decisions. **Results:** Experiments conducted on the newly curated LungCA dataset comprising 767 patients demonstrate that the model achieves 97.40% accuracy in the Turkish (TR) reporting scenario and 94.81% accuracy in the English (EN) scenario, alongside strong alignment with human-written reports in BLEU, ROUGE, METEOR, and CIDEr metrics. **Conclusions:** The findings demonstrate that the proposed multi-slice transformer framework achieves robust performance in both classification and radiology report generation, enhances transparency throughout the decision-making process, and provides a robust artificial intelligence solution capable of effectively supporting clinical workflows in lung cancer assessment.

## 1. Introduction

Cancer is a disease characterized by uncontrolled cellular proliferation and the disruption of normal cell death mechanisms [1]. Under physiological conditions, somatic cells maintain tissue integrity through a balanced cycle of growth and apoptosis [2]. During malignant transformation, however, cells lose control over proliferation, acquire resistance to programmed cell death, and gain the capacity for unchecked growth [3]. Despite major advances in molecular oncology, cancer remains one of the leading causes of morbidity and mortality worldwide [4,5]. According to recent global statistics, approximately 20 million new cancer cases and 9.7 million deaths were recorded in 2022 [6]. Among all malignancies, lung cancer ranks first, with an estimated 2.5 million new diagnoses and 1.8 million deaths, making it both the most common and the deadliest cancer type [6].

Lung cancer is histologically classified into two major categories: small-cell lung cancer (SCLC) and non-small-cell lung cancer (NSCLC) [7]. SCLC is characterized by an exceptionally high proliferative rate and a strong tendency for early hematogenous and lymphatic spread [8]. It typically presents as a centrally located mass near the main bronchi and is often accompanied by mediastinal lymph node enlargement and lung collapse secondary to airway obstruction [9]. Owing to its rapid progression and aggressive biology, SCLC is commonly diagnosed at an advanced or metastatic stage [10]. In contrast, NSCLC accounts for the majority of lung malignancies [11]. Unlike SCLC, it exhibits a comparatively slower growth pattern but demonstrates significant biological and morphological heterogeneity [12]. Radiologically, NSCLC often arises in the peripheral lung parenchyma and is subdivided into three principal histopathological types: adenocarcinoma, squamous cell carcinoma, and large cell carcinoma [13].

Adenocarcinoma is the most prevalent subtype and typically appears as a slowly growing peripheral lesion, yet possesses the capacity for early distant spread [14]. Squamous cell carcinoma shows a strong association with chronic tobacco exposure and commonly develops in the central airways through malignant transformation of the bronchial surface epithelium [15]. Because of its endobronchial localization, it often gives rise to early symptoms such as cough and hemoptysis [16]. Large cell carcinoma is relatively uncommon; however, its rapid growth and early metastatic behavior impart notable clinical significance [17].

These subtypes differ substantially in biological behavior, metastatic potential, therapeutic responsiveness, and overall prognosis [18]. Therefore, accurate subtype identification is essential for selecting the most effective treatment strategy. Biopsy remains the diagnostic gold standard, and its safe and efficient performance requires detailed knowledge of the tumor’s location, size, and relationship to surrounding structures [19]. At this stage, computed tomography (CT) imaging and radiology reports play an indispensable role. CT provides a clear depiction of tumor localization, delineates lesion size and margins, and guides selection of the optimal biopsy trajectory [20]. Radiology reports summarize these findings systematically, offering critical support to both the interventional team performing the biopsy and the clinical team responsible for treatment planning [21].

Manual evaluation of radiological images continues to be widely practiced in the diagnosis and follow-up of lung cancer [22]. However, it is labor-intensive, requires substantial expertise, demands considerable time, and contributes to a heavy clinical workload. A radiologist may need to examine thousands of CT slices individually to detect lesions and generate a comprehensive report. Increasing patient volumes and expanding clinical responsibilities further exacerbate these challenges [23]. In regions with limited access to experienced radiologists, interpretation times may be prolonged, leading to diagnostic delays. Moreover, manual image assessment is inherently subjective; identical radiological findings may be interpreted differently by different experts [24]. Such variability can reduce diagnostic accuracy, introduce uncertainty into treatment planning, and increase the risk of adverse patient outcomes.

Given these constraints, there is a growing need for new approaches that can enhance and standardize diagnostic workflows. Rapid advancements in digital radiology and artificial intelligence have provided a powerful foundation for addressing the limitations of manual image interpretation [25]. Deep learning-based automated image analysis systems are capable of processing large volumes of radiological data in a short time [26]. They can detect and classify lesions with high accuracy and consistency, thereby offering a supportive diagnostic layer for radiologists [27]. By reducing human error and inter-observer variability, these systems help improve diagnostic reliability. Building on these developments, numerous studies have focused on lung cancer classification using CT imaging. Selected examples of recent CT-based lung cancer classification studies are presented in Table 1.

In recent years, deep learning has initiated a significant methodological shift in medical image analysis and has rapidly become one of the field’s foundational approaches. Convolutional neural networks (CNNs), in particular, have been widely adopted in diagnostic systems due to their hierarchical feature extraction capabilities and their effectiveness in capturing local spatial patterns [37]. However, the inherently limited receptive field of convolutional layers constrains their ability to model broad contextual dependencies and fully represent complex anatomical structures [38]. This limitation becomes especially pronounced in high-dimensional medical imaging, where subtle or diffuse pathological patterns require a more comprehensive representation.

Transformer-based architectures have emerged as powerful alternatives in medical imaging [39]. Due to the global perceptual capacity provided by self-attention mechanisms, these models can effectively capture long-range dependencies and intricate contextual relationships across the entire image [40]. As a result, they have demonstrated substantial success in tasks that demand high-level spatial reasoning, including tumor classification [41], tissue segmentation [42], and lesion localization [43]. Nonetheless, high predictive performance alone is insufficient for artificial intelligence systems intended for clinical use. One of the key elements that enhances the reliability of medical decision-support models is explainability [44]. Beyond producing accurate results, an algorithm must also provide transparent insights into the visual or semantic cues on which its decisions are based. In radiological assessment, enabling experts to visualize the anatomical regions that inform the model’s predictions forms a critical bridge between technical success and clinical trust [45]. However, the majority of transformer-based studies targeting lung cancer focus primarily on performance metrics, while systematic treatment of explainability remains relatively limited.

Additionally, much of the existing research is confined to categorical classification frameworks that output a single class label or probability score. While informative, such outputs offer limited practical value in clinical decision-making. Radiologists evaluate lung cancer by integrating multidimensional findings into comprehensive, structured reports. Therefore, developing systems capable of generating coherent, linguistically consistent, end-to-end automated radiology reports from CT-derived visual representations remains a largely underexplored research direction. Moreover, many current approaches rely on single-slice analysis, which inadequately reflects the volumetric nature of CT data. A typical thoracic CT scan comprises hundreds of slices covering the entire lung volume, and accurate diagnostic evaluation requires extensive analysis including tumor delineation, assessment of invasion into surrounding tissues, and identification of metastatic lesions at multiple levels [46]. Consequently, classification methods that focus solely on a single slice fall short in terms of diagnostic completeness.

Despite extensive progress in CNN and transformer-based lung cancer classification, the literature on automated radiology report generation specifically for thoracic CT remains markedly limited. The majority of existing report-generation frameworks have been developed for chest X-ray imaging, where a single 2D projection is mapped to a textual description. Such designs cannot be directly transferred to CT, since thoracic CT is inherently volumetric and diagnostically meaningful findings are distributed across multiple axial levels. Recent CT-oriented transformer studies have begun to address slice-level reasoning, yet they remain restricted to classification and do not couple visual representations with clinically structured text output. As summarized in Table 1, none of the representative recent studies on lung cancer CT analysis couple multi-slice volumetric reasoning with native report generation, and explainability is treated only sporadically. The framework proposed in this study is positioned to fill exactly this gap by unifying multi-slice classification, bilingual radiology report generation, and slice-wise visual explanation within a single transformer pipeline.

The main contributions of this study are summarized as follows:

- A multi-slice volumetric encoding strategy is introduced. By jointly processing multiple CT slices, the model effectively learns spatial continuity and captures multidimensional anatomical relationships inherent in volumetric imaging.
- A novel feature aggregation mechanism, Learnable Query Attention Pooling (LQAP), is developed. By generalizing latent-query attention pooling to the multi-slice CT setting, LQAP compresses 985 inter-slice tokens (5 slices × 197 patch tokens) into a fixed 197-token patient-level embedding through a set of trainable latent queries, providing a content-adaptive alternative to mean, max, or single-head attention pooling.
- A cross-modal generative model is presented to convert visual information into clinically meaningful radiology reports. This approach provides an interpretable bridge between image-based perception and text-based diagnostic explanation.
- An explainability-centered transformer framework is introduced that generates attention maps consistent with true tumor regions, enabling clearer and clinically meaningful interpretation of model decisions.
- To the best of our knowledge, this study represents the first comprehensive multi-slice transformer-based framework for thoracic CT analysis that jointly performs classification, report generation, and explainability within a single unified system.

## 2. Materials and Methods

**Figure 1:**
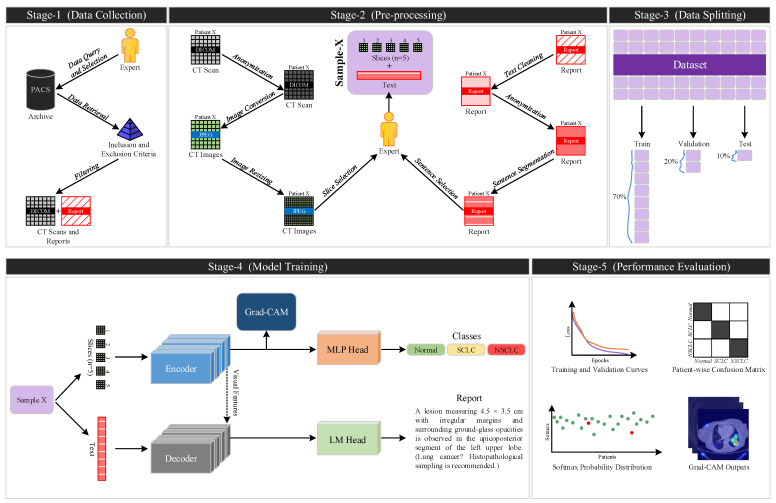
Methodological workflow of the proposed framework.

In this study, we present an end-to-end deep learning framework that classifies CT scans into three lung cancer categories (SCLC, NSCLC and Normal) while directly leveraging multi-slice imaging information. The proposed system employs transformer-based architectures to more effectively model volumetric anatomical structures and performs inference at the patient level. Furthermore, the model offers a high degree of explainability by enriching classification outputs with natural-language radiological descriptions and by providing interpretable visual attention maps that illuminate the underlying decision mechanisms. The overall methodological pipeline and the data flow between system components are summarized in Figure 1.

### 2.1. LungCA Dataset

The LungCA dataset used in this study is a retrospective clinical collection derived from thoracic CT examinations stored in the PACS archives of the Department of Radiology at Fethi Sekin City Hospital between 2019 and 2025. The data acquisition process was conducted in accordance with the ethical principles of the Declaration of Helsinki, and the study protocol received approval from the Firat University Ethics Committee (Approval No: 2024/13-38).

The selection of cases followed predefined inclusion and exclusion criteria to ensure methodological rigor and clinical homogeneity. Only thoracic CT scans from adult patients with diagnostic-quality imaging were included. For the lung cancer group, cases were required to demonstrate radiological evidence of a solid or subsolid malignant-appearing nodule or mass, with malignancy confirmed by histopathological diagnosis. The Normal category consisted of scans from patients without any history of malignancy and without active parenchymal pathology on CT imaging. Exclusion criteria included examinations affected by respiratory or motion artifacts, severe metal-induced distortion, or low signal-to-noise ratio. Cases involving secondary malignancy, metastatic lung involvement, or synchronous primary tumors were removed to avoid biological heterogeneity. Patients with incomplete clinical information or unverified radiology reports were also excluded to maintain methodological consistency.

All thoracic CT scans were performed on a Philips Ingenuity 128 CT device (Royal Philips Prinses, Amsterdam, The Netherlands). Imaging parameters were kept consistent with institutional standards, and all series were recorded at a resolution of 768 × 768 pixels with a slice thickness of 3 mm. Although the number of slices varied depending on patient anatomy, each examination contained approximately 700 axial slices on average.

**Figure 2:**
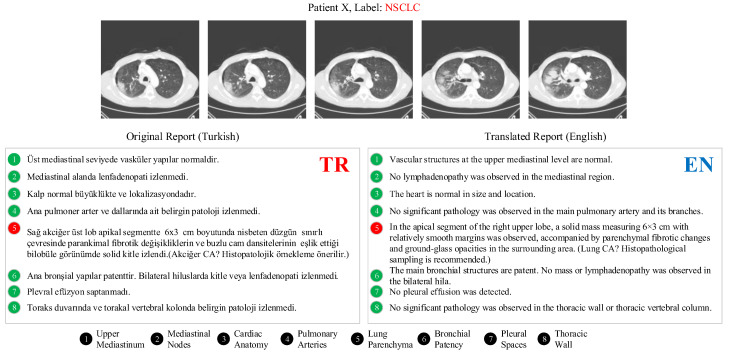
Sample CT scan and radiology report from the LungCA dataset.

In addition to patient imaging, the LungCA dataset includes corresponding radiology reports. Each report is an official clinical document generated during routine practice and follows an eight-sentence standardized structure. These reports provide a systematic evaluation of mediastinal, cardiac, pulmonary, bronchial, hilar, pleural, and thoracic wall structures, and describe parenchymal lesions in detail with respect to size, location, and morphological characteristics. The LungCA dataset comprises a total of 767 patients: 248 SCLC, 263 NSCLC, and 256 Normal cases. An example thoracic CT scan and its associated radiology report from the dataset are shown in Figure 2.

The LungCA dataset is inherently class-balanced: the three diagnostic categories are represented at proportions of 32.3% (SCLC), 34.3% (NSCLC), and 33.4% (Normal), corresponding to a maximum-to-minimum class ratio of approximately 1.06:1.00. This distribution falls well below the conventional thresholds used to define class imbalance and therefore does not require synthetic oversampling, class-weighted loss functions, or focal-loss reweighting strategies. To prevent any residual imbalance from propagating into the evaluation, patient-level stratified sampling was applied during the train/validation/test split (70%/20%/10%), ensuring that the original class proportions were preserved across all three subsets.

#### Annotation Protocol

Class labels (SCLC, NSCLC, Normal) were assigned through a two-stage protocol designed to combine pathological ground truth with radiological verification. For all 511 malignant cases, the diagnostic label was derived directly from the corresponding histopathological report obtained from biopsy, which served as the gold standard. Cases without histopathological confirmation were excluded from the malignant cohort. Normal cases were defined by the absence of any malignancy history and the absence of active parenchymal pathology on CT. Following label extraction, all CT volumes were independently re-reviewed by a board-certified thoracic radiologist with more than ten years of experience, who verified each label against the imaging findings through visual inspection of the full volumetric series. Cases for which the radiological appearance was inconsistent with the histopathological diagnosis were not encountered in the present cohort.

The same radiologist also performed the diagnostic slice selection that defines the 5-slice input used by the model. For malignant cases, the axial slice corresponding to the maximum axial tumor diameter was identified as the index slice, and the two cranially and two caudally adjacent slices were retained, yielding five contiguous axial slices per patient that fully cover the lesion volume. For Normal cases, in the absence of a focal lesion, five axial slices were selected at standardized anatomical landmarks representing the principal thoracic levels: (i) the lung apex, (ii) the aortic arch, (iii) the carinal bifurcation, (iv) the right lower lobe bronchus, and (v) the diaphragmatic dome. This systematic landmark-based scheme ensures comprehensive parenchymal coverage while preserving structural input consistency with the malignant cases. All slice selections were performed by the same radiologist to maintain inter-patient consistency.

### 2.2. Pre-Processing

Thoracic CT images were retrieved from the hospital’s PACS in DICOM format, with all 16-bit intensity information preserved during processing. In accordance with ethical requirements, all imaging data were irreversibly anonymized, and all metadata containing patient-identifiable information were removed. A clinically standardized preprocessing pipeline was applied to ensure compatibility with deep learning models and to improve computational efficiency. Each axial slice was windowed using parameters optimized for lung parenchyma visualization (Window Width: 1500 HU, Window Level: –600 HU), after which the images were rescaled to an 8-bit intensity range and converted from single-channel format to RGB. All images were then resampled to a resolution of 224 × 224 pixels using bilinear interpolation to satisfy model input size requirements. Processing the full volumetric series for each patient would be both computationally expensive and largely redundant for the present diagnostic task, since most axial slices contribute little incremental information beyond the lesion-bearing levels. Therefore, a diagnostic slice selection strategy was implemented. For each patient, five diagnostically representative axial slices were selected according to the protocol described in the Annotation Protocol subsection.

In parallel with image preprocessing, a structured NLP pipeline was applied to the associated radiology reports. Each report underwent anonymization, text cleaning, sentence segmentation, and content filtering to ensure linguistic and diagnostic consistency. First, all patient-identifying information was removed in accordance with ethical data handling protocols. Following anonymization, typographical errors were corrected and medical abbreviations were standardized. Only clinically meaningful sections of each report were retained, while administrative or secondary content was excluded. The original LungCA radiology reports consist of descriptive statements spanning eight thoracic anatomical categories; however, to improve computational efficiency and to ensure that the model focused on the most oncologically relevant information, only sentences corresponding to the “Lung Parenchyma” section were used, and all other categories were systematically excluded.

To evaluate the validity of the proposed framework both in its original language setting and across different linguistic environments, the selected parenchyma-focused statements were translated into English using professional medical terminology. Consequently, two experimental scenarios were constructed: an image–Turkish report pairing (Scenario 1) and an image–English report pairing (Scenario 2).

### 2.3. The Proposed Framework

**Figure 3:**
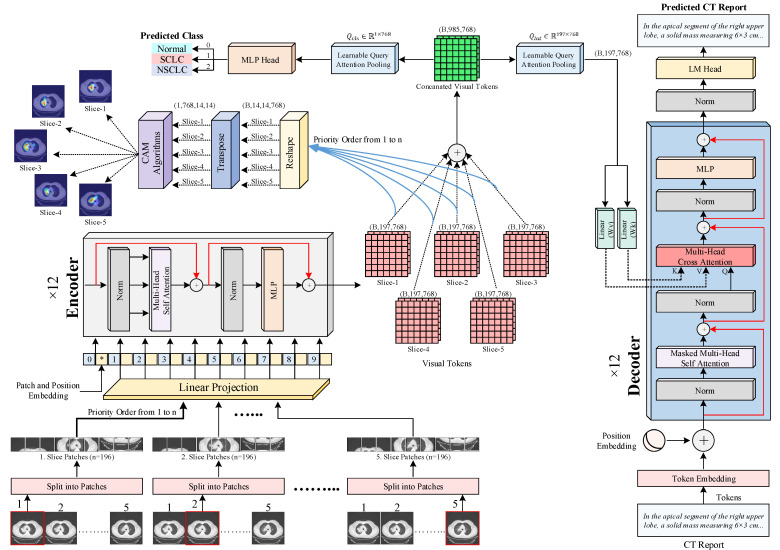
Architecture of the proposed multi-slice framework.

The proposed framework is an end-to-end transformer-based architecture designed for automated classification of thoracic CT images and generation of corresponding radiology reports. As illustrated in Figure 3, the framework comprises two main components operating within a unified processing pipeline. The first component is an encoder module inspired by the Vision Transformer (ViT) [47] architecture. This module extracts high-representational-capacity visual features from five CT slices belonging to the same patient. Simultaneous processing of multiple slices enables the model to capture contextual relationships between adjacent sections and to more effectively characterize structural and textural patterns. The second component is a GPT-2-based decoder architecture, which generates coherent and clinically meaningful radiology reports driven by the visual embeddings produced by the encoder.

#### 2.3.1. Multi-Slice Volumetric Input

In clinical settings, radiologists do not rely on a single CT slice when forming a diagnosis. Instead, they examine a series of neighboring slices to assess lesion continuity, spatial distribution, and the anatomical relationships of pathological findings. This sequential interpretation provides a more comprehensive understanding of disease patterns and supports accurate clinical decision-making. Inspired by this diagnostic approach, the proposed model employs a multi-slice input representation that mimics the reasoning process of human experts. For each patient, five CT slices are selected to construct the model input set as $S=\{I_{1},I_{2},I_{3},I_{4},I_{5}\}$.

Here, each slice $I_{i}\in R^{H\times W\times C}$ is standardized to a fixed spatial resolution of 224 × 224 pixels and represented as a three-channel image (H = W = 224, C = 3). The inclusion of multiple slices enhances the model’s ability to capture inter-slice continuity and regional coherence, enabling the encoder to form more stable and anatomically meaningful feature embeddings. Rather than relying solely on single-slice visual cues, the adopted representation facilitates a richer understanding of lesion boundaries, intensity transitions, and texture distributions across consecutive sections.

#### 2.3.2. Patch Tokenization and Positional Embedding

In accordance with the ViT paradigm [47], each input CT slice is decomposed into a set of fixed-size, non-overlapping image patches that serve as the fundamental visual tokens for transformer-based processing. This design strategy allows the model to treat medical images analogously to sequences of words in natural language processing, thereby bridging the gap between image understanding and sequential representation learning. For an input image with a spatial resolution of 224 × 224, each slice is partitioned into square patches of size *p* = 16 × 16, resulting in *n* = (224/16)2 = 196 non-overlapping patches per slice. Each patch represents a localized region of the image, capturing fine-grained anatomical details such as parenchymal texture, lesion boundaries, and density transitions that are diagnostically relevant in thoracic imaging. Each flattened patch $x_{p}^{i}\in R^{P^{2}\cdot C}$ is linearly projected into a D-dimensional embedding space (D = 768) through a learnable transformation (see Equation (1)).

(1) $$E_{p}x_{p}^{i}=\mathrm{Flatten}\left(x_{p}^{i}\right)\cdot W_{E}+b_{E}$$

where $W_{E}\in R^{\left(P^{2}\cdot C\right)\times D}$ denotes the patch projection matrix and $b_{E}\in R^{D}$ is a bias term. Through this projection, spatially localized intensity patterns are mapped into a continuous latent space that is jointly optimized during training to encode radiologically meaningful visual semantics. To preserve spatial ordering, a learnable positional embedding is added to each patch embedding. Since transformers are inherently permutation invariant, positional encodings provide the necessary spatial context. Furthermore, a dedicated classification token ${(x}_{cls})$ is prepended to the sequence, serving as a global summary representation.

The final embedded representation of a single slice, ready for the encoder, can thus be expressed as Equation (2).

(2) $$z_{0}=\left[x_{cls};E_{p}x_{p}^{1};E_{p}x_{p}^{2};\ldots ;E_{p}x_{p}^{N}\right]+E_{pos}$$

where $z_{0}\in R^{\left(N+1\right)\times D}$ is the input sequence for the encoder, and $E_{pos}\in R^{\left(N+1\right)\times D}$ is the learnable positional embedding matrix.

#### 2.3.3. Encoder Block

As depicted in Figure 3, the embedded patch sequence obtained from each CT slice is subsequently processed by a multi-layer ViT encoder composed of twelve identical transformer blocks. Each block performs hierarchical feature transformation through self-attention mechanisms, enabling the model to learn long-range dependencies and capture both local and global contextual cues within the slice. Each encoder block consists of two primary sublayers: a multi-head self-attention (MHSA) mechanism and a feed-forward multilayer perceptron (MLP). Both sublayers are wrapped with residual connections and layer normalization (LN).

For a given input sequence $z_{l-1}$ entering the l-th encoder layer, the computation proceeds as follows:

(3) $$z_{l}^{\prime }=\mathrm{MHSA}\left(\mathrm{LN}\left(z_{l-1}\right)\right)+z_{l-1}$$

(4) $$z_{l}=\mathrm{MLP}\left(\mathrm{LN}\left(z_{l}^{\prime }\right)\right)+z_{l}^{\prime }$$

In the self-attention mechanism, each token interacts with every other token through learned query (Q), key (K), and value (V) projections. The attention weights are computed as

(5) $$\mathrm{Attention}\left(Q,K,V\right)=\mathrm{softmax}\left(\frac{QK^{T}}{\sqrt{d_{k}}}\right)V$$

here $d_{k}$ is the dimensionality of the key vectors, serving as a normalization factor. By employing multiple attention heads (h = 12), the model can jointly attend to information from different representational subspaces. After the twelfth layer, the encoder outputs a contextualized token sequence for each slice, which serves as the foundation for inter-slice aggregation.

#### 2.3.4. Cross-Slice Aggregation with LQAP

Inspired by diagnostic reasoning, the framework aggregates visual features from all five slices into a unified latent representation. This step models three-dimensional anatomical coherence across the stack of slices. Each slice-level encoder output $z_{L}^{\left(i\right)}\in R^{\left(N+1\right)\times D}$ contains 197 contextualized tokens.

For a patient represented by M = 5 slices, these feature sequences are concatenated along the token dimension:

(6) $$X_{concat}=\mathrm{Concat}\left[z_{L}^{\left(1\right)},z_{L}^{\left(2\right)},\ldots ,z_{L}^{\left(5\right)}\right]\in R^{\left(M\cdot \left(N+1\right)\right)\times D}$$

The resulting sequence of 985 tokens is computationally intensive. To derive a compact representation, a learnable query-based latent pooling mechanism is applied. A set of trainable latent vectors $Q_{lat}\in R^{N_{lat}\times D}$ acts as abstract queries that attend to the concatenated token space. Here, $N_{lat}$ is set to 197 for structural consistency. The pooling operation is formulated as

(7) $$Z_{latent}^{\prime }=\mathrm{MHA}\left(\mathrm{LN}\left(Q_{lat}\right),\mathrm{LN}\left(X_{concat}\right),\mathrm{LN}\left(X_{concat}\right)\right)+Q_{lat}$$

To further refine the pooled representation, a feed-forward MLP is applied:

(8) $$Z_{latent}=\mathrm{MLP}\left(\mathrm{LN}\left(Z_{latent}^{\prime }\right)\right)+Z_{latent}^{\prime }$$

The output $Z_{latent}\in R^{197\times D}$ serves as the final multi-slice latent embedding. This compact representation encapsulates both spatial structure within slices and volumetric continuity across them, providing a semantically consistent foundation for downstream tasks.

The motivation for introducing a learnable query-based pooling mechanism, rather than relying on simpler alternatives, lies in the unique structure of multi-slice CT representations. After processing five axial slices through the ViT encoder, the resulting 985-token sequence contains both clinically informative tokens and large numbers of clinically less informative tokens. A simple mean pool would assign uniform weight to every position and dilute the diagnostic signal contributed by the small subset of lesion-relevant patches. Max pool preserves the strongest activation per feature dimension but discards distributional context. A single-head attention pool, where one learnable query attends to all tokens, addresses these concerns partially but compresses the entire 985-token space into a single weighted average and therefore offers only one perspective of the input. LQAP generalizes this last idea by introducing multiple trainable latent queries, each free to attend to a different aspect of the multi-slice representation, producing a content-adaptive patient-level embedding that aggregates clinically informative content across the slice stack independently of slice ordering.

Implementation-wise, both the inter-slice latent queries (197 × 768) and the patient-level class query (1 × 768) are realized as tensors initialized with Xavier uniform distribution. Each LQAP block follows a pre-norm Transformer design: query, key, and value tensors are passed through independent LayerNorm layers prior to the multi-head attention operation (12 heads, head dimension 64), and residual connections are applied after both the attention and the feed-forward (MLP expansion ratio 4×, GELU activation, dropout 0.1) sub-modules. The two LQAP modules together contribute 14.33 M trainable parameters, corresponding to approximately 5.7% of the full model.

#### 2.3.5. Patient-Level Classification Head

To emulate how radiologists synthesize observations into a single conclusion, a patient-level pooling mechanism condenses the multi-token embedding $Z_{latent}$ into a single feature vector representing the integrated patient state. A learnable query token, $Q_{cls}\in R^{1\times D}$, is used to perform attention pooling over the latent feature sequence.

This token attends to all embeddings in $Z_{latent}$ to gather the most diagnostically relevant signals:

(9) $$z_{patient}^{\prime }=\mathrm{MHA}\left(\mathrm{LN}\left(Q_{cls}\right),\mathrm{LN}\left(Z_{latent}\right),\mathrm{LN}\left(Z_{latent}\right)\right)+Q_{cls}$$

To enhance its discriminative capacity, the resulting vector passes through another MLP:

(10) $$z_{patient}=\mathrm{MLP}\left(\mathrm{LN}\left(z_{patient}^{\prime }\right)\right)+z_{patient}^{\prime }$$

This final feature descriptor $z_{patient}\in R^{D}$ serves as input to the classification head, which maps the patient embedding to one of the diagnostic categories. The model is configured for a three-class classification task. The head predicts logits for three classes corresponding to Normal, SCLC, and NSCLC:

(11) $${\mathrm{Logits}}_{cls}=\mathrm{Linear}\left(\mathrm{GELU}\left(\mathrm{Linear}\left(\mathrm{LN}\left(z_{patient}\right)\right)\right)\right)\in R^{3}$$

The classification loss is computed using the standard cross-entropy function, which is suitable for multi-class problems:

(12) $$L_{cls}=\mathrm{CrossEntropy}\left(\mathrm{Softmax}\left({\mathrm{Logits}}_{cls}\right),y_{true}\right)$$

#### 2.3.6. Decoder and Cross-Modal Conditioning

The framework incorporates a GPT-2-based text decoder to generate radiology reports conditioned on the visual embeddings. The decoder consists of twelve transformer blocks, each comprising three sublayers: masked self-attention, cross-attention, and a feed-forward network.

The integration between the visual encoder and the textual decoder is realized through a shared 768-dimensional latent space, which ensures direct compatibility without requiring an additional projection layer. After the LQAP modules produce the patient-level visual context tensor of shape (B, 197, 768), this tensor is passed unchanged to every decoder block as the source of cross-attention keys and values. The textual side, in contrast, supplies the queries: each token embedding is projected into a query vector inside the cross-attention sublayer. Consequently, every token being generated attends to all 197 visual tokens of the LQAP context, and the resulting attention distribution determines which anatomical regions inform the next word. This single shared embedding dimensionality is what allows the visual evidence accumulated by the ViT–LQAP encoder to flow seamlessly into the autoregressive language-modeling pipeline of the decoder. A summary of the tensor shapes and roles across this pipeline is provided in Table 2.

The input text sequence is first embedded via the sum of token and positional embeddings:

(13) $$h_{0}=E_{token}+E_{pos}$$

Within each decoder block l, the textual representation $h_{l-1}$ is sequentially refined. First, a masked multi-head self-attention layer processes the text, where a causal mask prevents tokens from attending to future positions, ensuring autoregressive generation:

(14) $$h_{l}^{\prime }=\mathrm{Masked}\text{-}\mathrm{MHSA}\left(\mathrm{LN}\left(h_{l-1}\right)\right)+h_{l-1}$$

Next, a cross-attention layer integrates visual context. It uses the text representation $h_{l}^{\prime }$ as the query and the multi-slice latent embedding $Z_{latent}$ as the key and value. This allows each text token to dynamically attend to the most relevant visual features:

(15) $$h_{l}^{\prime \prime }=\mathrm{CrossAttention}\left(\mathrm{LN}\left(h_{l}^{\prime }\right),Z_{latent},Z_{latent}\right)+h_{l}^{\prime }$$

Finally, an MLP layer further processes the fused representation:

(16) $$h_{l}=\mathrm{MLP}\left(\mathrm{LN}\left(h_{l}^{\prime \prime }\right)\right)+h_{l}^{\prime \prime }$$

After the final decoder block, a linear block (the LM Head) projects the output sequence $h_{12}$ to the vocabulary size to produce logits for the next token prediction. The report loss is also calculated using cross-entropy:

(17) $$L_{report}=\mathrm{CrossEntropy}\left({\mathrm{Logits}}_{txt},y_{report}\right)$$

Two complementary regimes govern how the decoder consumes the visual context. During training, the model operates under a teacher-forcing protocol: the entire ground-truth report is fed in parallel as input tokens, the causal mask in the masked self-attention sublayer prevents each position from peeking at future tokens, and the cross-attention sublayer of every block conditions all positions on the same LQAP context tensor, allowing the language-modeling loss in Equation (17) to be computed jointly across the full sequence in a single forward pass. During inference, generation proceeds autoregressively: the sequence is initialized with the GPT-2 beginning-of-sequence token and the model emits one token at a time. At each step, the newly generated token is appended to the input, the decoder is re-evaluated, and the cross-attention sublayers continue to attend to the same fixed LQAP context until the end-of-sequence token is produced.

#### 2.3.7. Explainability with Grad-CAM Visualization

To enhance the interpretability and diagnostic transparency of the multi-slice transformer framework, a slice-specific gradient-based attribution strategy was employed to visualize the spatial evidence that most strongly guided the model’s classification decisions. In this setting, five consecutive axial CT slices were independently processed through the encoder, yielding a set of contextualized token representations, each corresponding to a distinct anatomical level of the thorax.

For each slice, the gradient of the target class logit with respect to the attention map $A^{k,\left(i\right)}$ from the final self-attention block was computed, and spatial importance weights were obtained as

(18) $$\alpha_{k}^{c,\left(i\right)}=\frac{1}{Z}\sum_{u}\sum_{v}\frac{\partial y^{c}}{\partial A_{uv}^{k,\left(i\right)}}$$

The Grad-CAM attention for slice i and class c was then derived according to

(19) $$L_{\mathrm{Grad}\text{-}\mathrm{CAM}}^{c,\left(i\right)}=\mathrm{ReLU}\left(\sum_{k}\alpha_{k}^{c,\left(i\right)}A^{k,\left(i\right)}\right)$$

producing a distinct saliency distribution for each of the five slices. Because the encoder represents local features as flattened patch tokens, the token embeddings were spatially reconstructed through a reshape operation $R_{\mathrm{attn}}\left(z_{L}^{\left(i\right)}\right)\in R^{14\times 14\times D}$, restoring the anatomical geometry of the original image grid. Each slice-specific Grad-CAM map was up-sampled to 224 × 224 resolution and superimposed on its respective CT slice, enabling a direct visualization of the spatial attention patterns underlying the model’s inference.

## 3. Results

### 3.1. Experimental Setup

All experiments were conducted on a high-performance workstation equipped with an NVIDIA RTX 4090 GPU and CUDA support, using the PyTorch (v2.5.1) framework. To ensure reproducibility, all sources of randomness were controlled through fixed seed values, and data splitting was performed at the patient level to prevent slices from the same individual from appearing in different subsets. The LungCA dataset was divided into training (70%), validation (20%), and test (10%) subsets while preserving class balance across all partitions. The hyperparameters used for the components of the proposed framework are summarized in Table 3.

#### Training Strategy

The proposed framework was trained end-to-end without freezing any sub-network. The ViT encoder was initialized from publicly available ImageNet-pretrained weights (vit_base_patch16_224 from the timm library), while the GPT-2 decoder was initialized from language-specific pretrained checkpoints: gpt2 from HuggingFace for the English scenario and gorkemgoknar/gpt2-small-turkish for the Turkish scenario. Both decoder checkpoints share the 50,257-token GPT-2 byte-pair-encoding vocabulary. The matrix shapes that match between the pretrained checkpoints and our custom decoder are transferred during initialization, while the cross-attention layers, which are unique to our multi-modal architecture and therefore absent from the original GPT-2, are trained from scratch. The token-embedding matrix and the language-modeling head share weights (weight tying) to reduce parameter count and stabilize training dynamics. In total, the model contains approximately 253.13 M trainable parameters, all of which are jointly optimized.

During training, early stopping (patience = 5) and validation-based model checkpointing were employed to prevent overfitting. The optimization process was carried out using the Adam optimizer combined with a OneCycleLR schedule (max learning rate = 1 × 10^−4^, with the built-in 30% warmup ratio followed by cosine annealing over the full training horizon), and mixed-precision training (PyTorch AMP (v2.5.1) with GradScaler) was applied to improve computational efficiency without sacrificing numerical stability. The batch size was set to 16, and the model was trained for a maximum of 100 epochs in each scenario. Since the model simultaneously performs two tasks, training was optimized using a multi-objective loss function. The total loss function is defined in Equation (20).

(20) $$L_{\mathrm{total}}=L_{\mathrm{cls}}+\lambda L_{\mathrm{report}}$$

Here, $L_{\mathrm{cls}}$ denotes the cross-entropy loss for three-class lung cancer classification, while $L_{\mathrm{report}}$ represents the language modeling loss for automatic report generation. Based on preliminary validation experiments, the weighting factor was set to λ = 0.3, ensuring a balanced contribution from both tasks during learning.

Beyond the optimization protocol described above, two further considerations governed the training of the language-modeling component, both of which deserve explicit clarification. Because radiology reports differ stylistically and lexically from general web text, the use of a general-purpose language model raises legitimate questions about domain adaptation. We deliberately chose not to substitute a biomedical-domain language model for two principal reasons. First, no comparable domain-pretrained Turkish counterpart is publicly available, and using such a model only for the English scenario would have introduced an asymmetric bilingual setting and prevented the like-for-like comparison that motivated the dual-language design of this study. Second, our architecture compensates for the absence of biomedical pre-training through two complementary mechanisms intrinsic to the framework: the cross-attention sublayers anchor every generated token to the multi-slice CT evidence at every decoder block, providing strong visual grounding that constrains the model toward radiologically plausible outputs; and the joint optimization with the classification loss acts as a domain-aware regularizer that biases the shared 768-dimensional latent space toward features that are diagnostically discriminative.

The decoders retain the standard 50,257-token GPT-2 byte-pair-encoding (BPE) vocabulary in both scenarios, with no domain-specific tokens added. Specialized radiological terms that are not present as single tokens in the base vocabulary are handled through BPE’s natural subword decomposition: such terms are split into smaller fragments at the input stage and re-assembled into coherent expressions through the contextual representations learned during fine-tuning. Because the language-modeling loss is computed jointly over all sub-token positions of each clinical expression, the decoder learns to reproduce these recurring fragment patterns with high fidelity.

### 3.2. Evaluation Metrics

Performance evaluation was conducted using a comprehensive set of metrics applied to both the image-based classification outputs and the text-based report generation results. For the three-class classification task, diagnostic discrimination ability was assessed through Accuracy, Recall, Precision, and F1-Score. For the automatic report generation task, the similarity between the generated textual outputs and the corresponding clinical reports was quantitatively evaluated using BLEU-1/2/3/4, ROUGE-L, METEOR, and CIDEr metrics. These measures enabled a comparative analysis of the model’s semantic accuracy, content consistency, and terminological alignment.

To further support explainability, visual interpretation of attention maps was performed, and the relationship between the anatomical regions attended by the model and the actual locations of pathological lesions was examined qualitatively. All evaluation metrics and their mathematical formulations used in this study are summarized in Table 4.

### 3.3. Experimental Results

**Figure 4:**
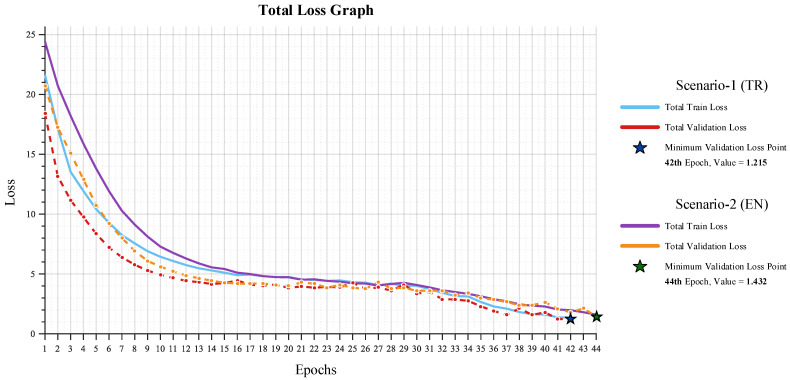
Training and validation loss curves.

Throughout the training process, loss curves for both scenarios were monitored, and the model achieving the lowest validation loss was automatically saved as the best-performing checkpoint. The training and validation loss curves of the proposed framework for each scenario are presented in Figure 4.

The overall training and validation losses obtained for both scenarios indicate that the model exhibits a stable and well-balanced convergence behavior. In the English configuration, the validation loss reached its minimum value of 1.432 at epoch 44, whereas in the Turkish configuration, the lowest validation loss of 1.215 was recorded at epoch 42. The close and parallel progression of the training and validation curves across all epochs suggests that the learning process did not deviate toward either overfitting or underfitting, and that the classification and report-generation tasks were jointly optimized in a coherent manner. This consistent convergence pattern further confirms that gradient flow remained stable throughout training, with no signs of oscillation, divergence, or stagnation.

#### 3.3.1. Visual Classification Results

**Figure 5:**
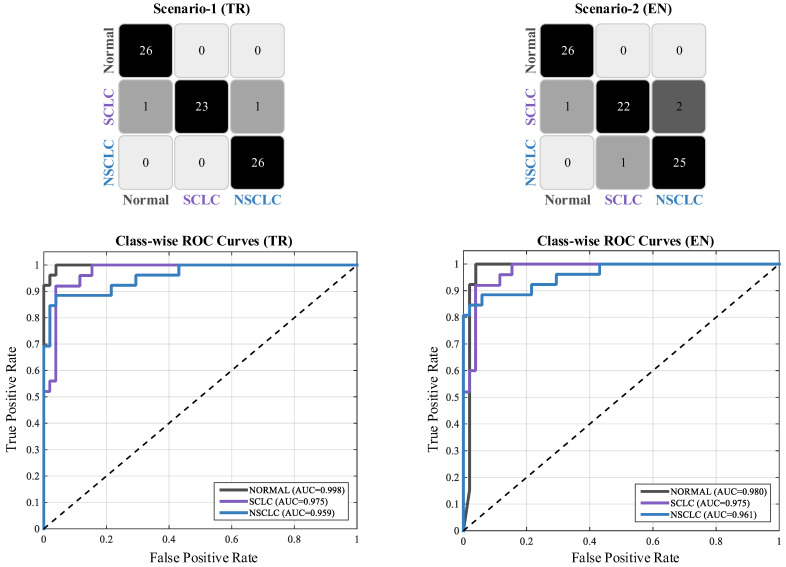
Confusion matrices and ROC curves at the patient level.

The visual classification performance of the proposed multi-slice transformer architecture was comprehensively evaluated through both quantitative metrics and qualitative analyses. All assessments were conducted on an independent test subset comprising 10% of the dataset to objectively validate the model’s generalization capability. Figure 5 presents the patient-level confusion matrices and class-wise ROC curves, illustrating the model’s ability to discriminate among the three categories.

For the three-class lung cancer classification task, the Turkish configuration demonstrated a notably high discriminative capacity. Analysis of the confusion matrix revealed that all Normal and all NSCLC cases were correctly classified, indicating that the model established an error-free decision boundary for these two categories. Within the SCLC class, only two misclassifications were observed—one instance predicted as Normal and one as NSCLC. The class-wise ROC curves displayed nearly ideal trajectories approaching the upper-left corner, and the resulting high AUC values (Normal: 0.998, SCLC: 0.975, NSCLC: 0.959) further supported the model’s strong discriminative performance.

The English configuration similarly exhibited robust classification behavior. In this setting, all Normal cases were correctly identified, and 25 out of 26 NSCLC cases were accurately classified. Three misclassifications occurred in the SCLC category: two were incorrectly assigned to NSCLC and one to Normal. Scenario 2 achieved an overall accuracy of 94.81%, and the high AUC values (Normal: 0.980, SCLC: 0.975, NSCLC: 0.961) confirmed the model’s reliable performance across classes.

Across both scenarios, the consistently high results clearly indicate that the model is capable of accurately distinguishing among lung cancer subtypes. Table 5 summarizes the quantitative classification metrics derived from the confusion matrices. In addition to precision, recall and F1-score, per-class and macro-averaged specificity values are reported, demonstrating that the model maintains very low false-positive rates across all three categories: macro-specificity reaches 98.69% in the Turkish scenario and 97.40% in the English scenario, with the lowest single-class value being 96.08% (NSCLC, English).

**Figure 6:**
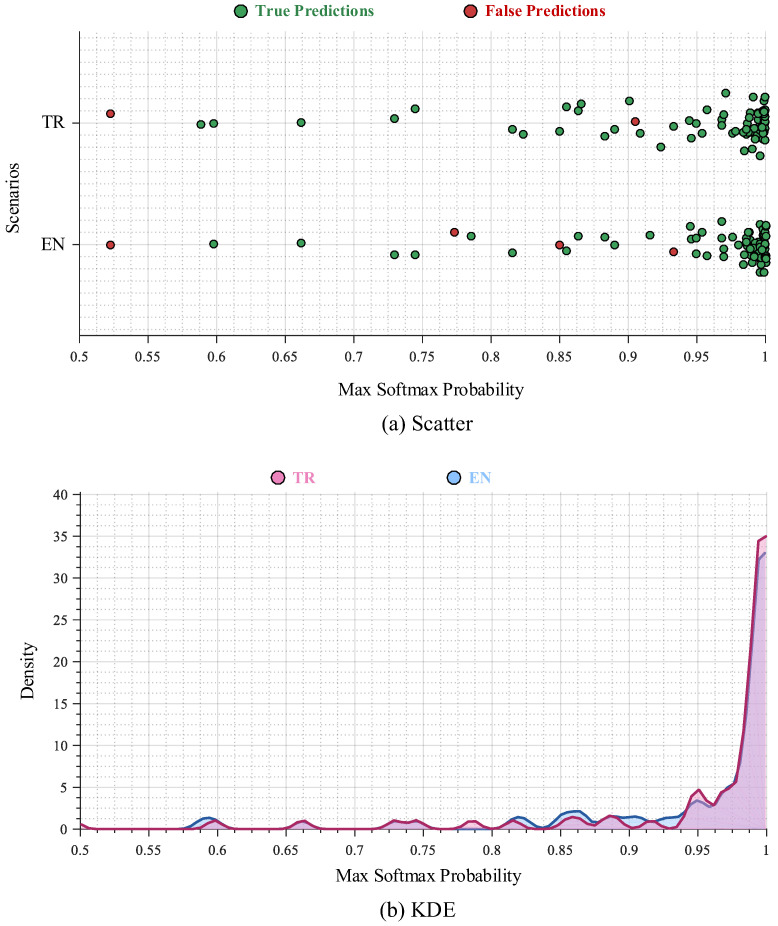
Distribution of maximum softmax probabilities across both scenarios: (**a**) scatter plots for correct and incorrect predictions; (**b**) KDE curves showing confidence distributions.

To more thoroughly characterize the model’s decision-making behavior and examine the structure of its confidence levels, the distribution of maximum softmax probabilities for correctly and incorrectly classified cases was analyzed across both experimental scenarios. The resulting distributions are visualized in the scatter plots and kernel density estimation (KDE) graph shown in Figure 6.

The distributions presented in the plots indicate that, in both scenarios, correctly classified instances are predominantly concentrated within the 0.90–1.00 probability range. This clustering in the high-confidence region demonstrates that the model establishes a wide decision margin for accurate predictions, maintains low uncertainty, and calibrates its confidence scores in a stable manner. The tendency of softmax values to lie near the upper bound suggests that samples belonging to the correct class are well-separated from the decision boundary, reflecting limited epistemic and aleatoric uncertainty. In contrast, misclassified samples cluster mainly within the 0.50–0.93 interval, indicating that errors arise from cases positioned near class boundaries or from samples with inherently low discriminative cues. The density observed in this mid-probability band shows that the model appropriately reflects uncertainty in its confidence scores and produces lower confidence levels for ambiguous or weakly separable cases. The absence of an overconfidence pattern further confirms that the decision mechanism behaves cautiously in high-risk regions and that its probabilistic outputs are structurally well-calibrated. The observed distributional pattern reveals a clear separation between high-confidence correct predictions and lower-confidence errors. This consistent distinction highlights the geometric stability of the decision function, the reliability of probabilistic calibration, and the model’s strong class discrimination capabilities. The orderly structure of the confidence scores indicates that the system provides not only high accuracy but also stable decision reliability.

**Figure 7:**
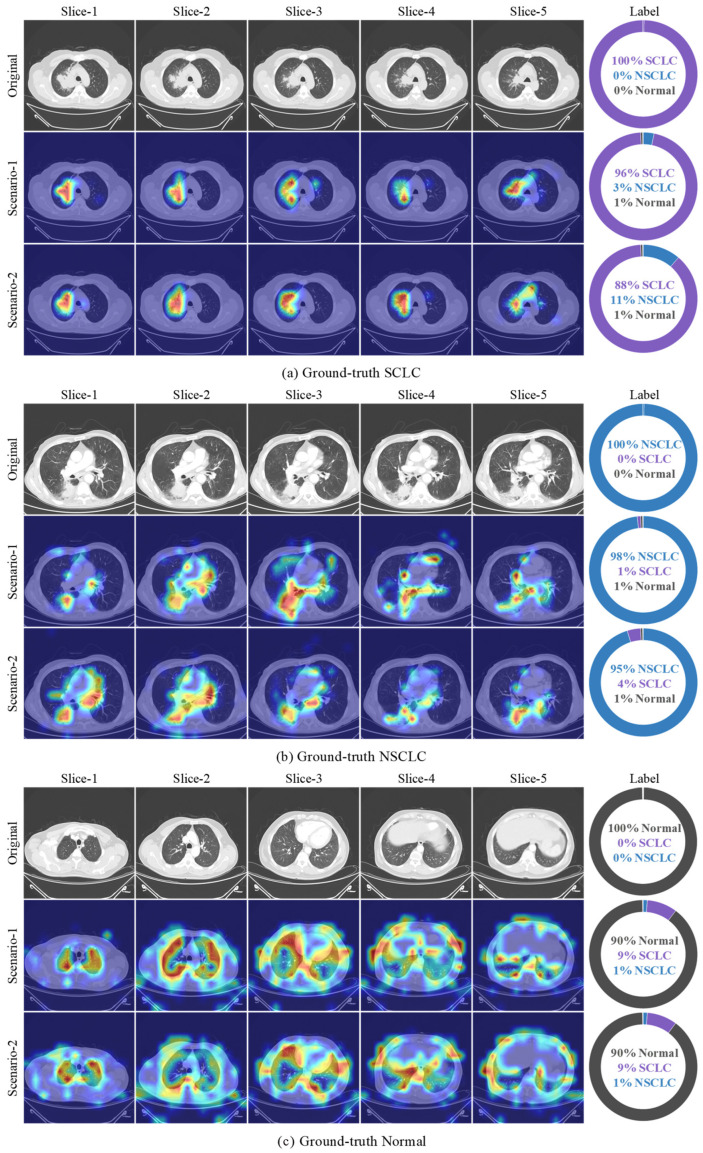
Slice-wise Grad-CAM visualizations across both scenarios: (**a**) SCLC, (**b**) NSCLC, and (**c**) Normal. The heatmaps indicate the model’s focus areas, where red represents high activation (importance) and blue represents low activation.

Having established that the model’s confidence structure is stable and well-calibrated based on the distribution of maximum softmax probabilities, it becomes essential to examine the visual evidence underlying these confidence levels. For a high-confidence prediction to hold methodological value, the model must derive its decision from anatomically or pathologically meaningful regions. Therefore, Grad-CAM outputs were analyzed to identify the spatial features emphasized by the model and to evaluate the alignment between predictions and supporting evidence. Representative samples from each class, randomly selected from the test set, together with their corresponding class predictions and attention regions, are shown in Figure 7.

The projection of Grad-CAM attention maps onto the multi-slice CT images demonstrates that, for both Scenario 1 (TR) and Scenario 2 (EN), the model maintains a structure capable of providing high diagnostic accuracy while preserving a spatially meaningful attention mechanism. In both configurations, the visualizations indicate that the classification outputs extend beyond a purely statistical probability estimation and that the decisions are consistently grounded in anatomically and pathologically informative regions. A detailed expert interpretation for each case presented in Figure 7 is given below:

For the SCLC case (Figure 7a), in the axial slices, the Grad-CAM outputs produced under both Scenario 1 and Scenario 2 show that the model is able to capture prominent cues characteristic of highly malignant, infiltrative, and rapidly proliferative tumor morphology. Attention intensities reach their maximum particularly in the hilar–perihilar region, over solid and irregular-density areas adjacent to broncho-vascular structures. This focus pattern is consistent with the clinically known central localization tendency of SCLC and its peribronchial invasion. The limited variation in attention locations across slices indicates that the model forms a continuous attention distribution that correctly represents the compact volumetric structure of the mass. In Scenario 2, the sharper attentions that more clearly align with the solid components suggest that the model produces a more focused response to regions exhibiting high cellularity and low internal heterogeneity, which are typical of aggressive tumor tissue. In contrast, the broader attentions observed in Scenario 1, extending into surrounding tissues, suggest that the model also evaluates peripheral morphological cues such as bronchial narrowing, mucosal irregularity, or subtle infiltrative findings. This distribution indicates that the classification decision is based not only on the geometric presence of the mass but also on the centrally located infiltrative growth dynamics characteristic of SCLC.

For the NSCLC case (Figure 7b), the attention patterns demonstrate that the model is sensitive both to the heterogeneous internal architecture of the lesion and to reactive parenchymal changes observed in the tumor microenvironment. Across all five slices, high attention levels concentrate on the heterogeneous solid mass in the right lower lobe, ground-glass components, perinodular infiltrations, and the volumetric variation regions defining the posterior–inferior extension of the tumor. In Scenario 1, the multifocal, fragmented, and widespread attention patterns reveal that the model is highly responsive to the biologically complex morphology of NSCLC, including solid–ground-glass transition zones, spiculated margins, interstitial thickening, and peribronchial reaction. In Scenario 2, attention clusters more clearly on the dense solid segments of the lesion, indicating a more selective discriminative tendency toward high-attenuation malignant components. In both scenarios, the spatial continuity of attentions across slices supports that the model internalizes the multi-slice volumetric pattern of the tumor rather than relying on a single slice. This finding shows that the model correctly captures the heterogeneous and peripherally infiltrative growth characteristics that are clinically critical for NSCLC.

For the Normal case (Figure 7c), the Grad-CAM outputs are characterized by low-intensity and non-focal attentions, as expected when distinguishing non-pathological lung morphology. In both scenarios, the diffuse distribution of low-density attentions indicates that the model does not base its classification on any specific pathological focus. This is an important indicator of a low false-positive tendency. Although Scenario 1 occasionally shows minimal attention reflections near subpleural or broncho-vascular structures, these signals remain consistently low-level and clinically insignificant across slices, demonstrating that the model does not misinterpret normal variations as pathological. In Scenario 2, the more homogeneous and low-contrast attentions reflect the high structural consistency of normal parenchyma, strongly supporting the model’s decision for the “negative class.” The attention behavior observed in the Normal class confirms that the model correctly represents normal lung anatomy and produces class-specific attention patterns only in the presence of pathology.

To complement the qualitative Grad-CAM analysis presented in Figure 7, a quantitative validation of the saliency maps was performed against expert-annotated tumor regions. The same board-certified thoracic radiologist who produced the dataset labels also annotated bounding boxes for the tumor in every available slice of all 51 malignant test patients (25 SCLC and 26 NSCLC), yielding 255 annotated slices in total. For each slice, the upper 20% activation region of the Grad-CAM map was converted to a binary mask and compared with the radiologist’s bounding box. The intersection-over-union (IoU), Dice similarity, and Pointing Game accuracy (the proportion of cases in which the single highest-activation pixel falls inside the annotation) were computed across all annotated slices. As summarized in Table 6, the model achieves a mean IoU of 0.45 ± 0.14, a mean Dice of 0.59 ± 0.15, and a Pointing Game accuracy of 85.4%, demonstrating that the attention patterns produced by the model are not merely visually plausible but also quantitatively aligned with the spatial regions identified by an expert radiologist as diagnostically relevant. The slightly higher localization scores observed for the SCLC class (IoU 0.48 versus 0.42 for NSCLC) are consistent with the typically more centrally located, structurally compact morphology of small-cell lesions, in contrast to the more peripheral and heterogeneous appearance of non-small-cell tumors.

#### 3.3.2. Report Generation Results

In addition to evaluating the performance of the visual classification component, the accuracy and consistency of the automatic diagnostic report generation module were also analyzed. At this stage, the primary focus is on the ability of the language modeling component to transform the high-dimensional representation space extracted by the multi-slice visual encoder into clinically valid, context-aware, and semantically coherent textual reports. As part of the quantitative analysis, the lexical and semantic similarity between the model-generated reports and the original official reports was assessed using standard metrics such as BLEU, ROUGE, and METEOR. The performance outputs obtained for Scenario 1 (TR) and Scenario 2 (EN) are presented in detail in Table 7.

The results clearly demonstrate that the model achieves high accuracy in clinical report generation. In the Turkish scenario, BLEU-1, BLEU-2, and BLEU-3 scores of 0.512, 0.413, and 0.316, respectively, indicate that the model can reproduce short and medium-length diagnostic n-gram patterns with high precision. Similarly, the English scores of 0.509, 0.389, and 0.295 show that the performance difference between the two languages is limited to approximately 6–7%, suggesting that the model preserves structural n-gram consistency to a large extent regardless of language. The BLEU-4 scores (0.233 for Turkish and 0.235 for English) being nearly identical further indicate that longer diagnostic expression structures are reproduced with similar accuracy in both languages. The ROUGE-L values of 0.574 (TR) and 0.514 (EN) reveal an approximately 11.7% advantage in favor of the Turkish outputs. This difference indicates that Turkish reports achieve higher sequential overlap with the original texts and that the model maintains narrative flow more strongly in Turkish. METEOR scores of 0.272 (TR) and 0.254 (EN) show that semantic and stem-level alignment is stable in both languages, though Turkish retains an approximate 7% advantage. The CIDEr scores were reported as 0.493 for Turkish and 0.442 for English. This 11.5% difference indicates that rare but diagnostically critical expressions are captured more accurately in Turkish.

**Figure 8:**
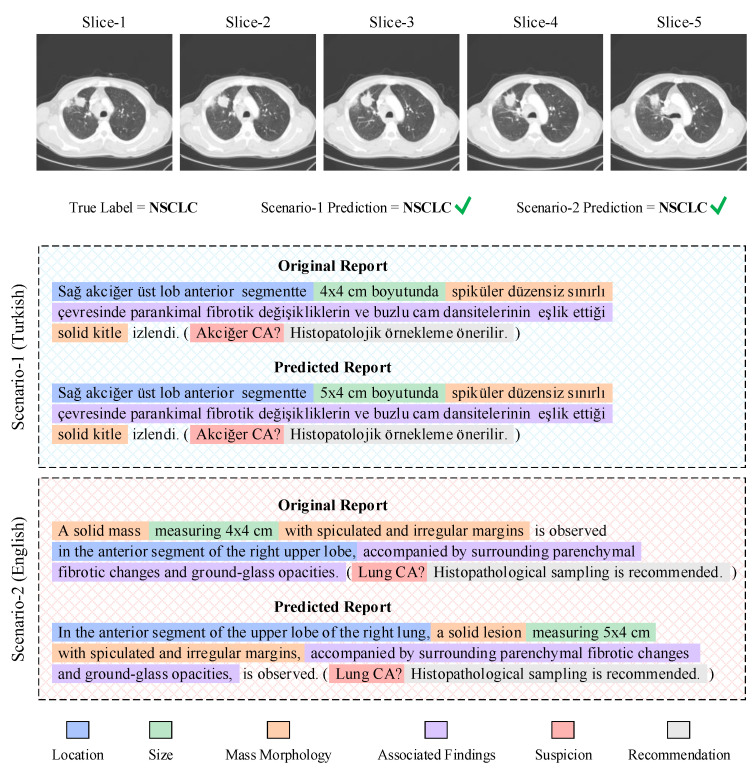
Original and predicted reports for both scenarios with semantic highlights. Check marks indicate correct predictions.

A comprehensive analysis of these scores suggests that the shared semantic representations produced by the multi-slice visual encoder can be transferred into text with high accuracy in both languages; however, the Turkish scenario demonstrates a noticeable advantage, particularly in ROUGE-L and CIDEr metrics. To examine the qualitative reflections of this quantitative trend, an example output from a randomly selected patient chosen from among the malignant classes is presented in Figure 8.

When the report generation outputs for both Scenario 1 (TR) and Scenario 2 (EN) are examined together, it becomes evident that the model can transform multi-slice visual representations into textual descriptions with high consistency in both languages. In both configurations, the system successfully reproduces the core components of radiological reporting within the correct hierarchical structure. The accurate transfer of anatomical location, preservation of size information, correct clinical terminology for morphological features such as spiculated or irregular contours, and contextually coherent representation of accompanying findings such as fibrotic changes or ground-glass opacities collectively demonstrate that the visual encoder can extract semantically stable and highly discriminative representations from volumetric lung data. The ability of the language model to convert these representations into clinically valid, contextually appropriate, and terminologically consistent reports strongly reflects the effectiveness of the system’s cross-modal information transfer.

In the English report generation setting, the model exhibits a tendency toward linguistic restructuring that optimizes the text structurally and syntactically while preserving the underlying clinical content. The placement of anatomical localization at the beginning of the sentence in the predicted reports aligns with the commonly used “location-first” narrative format in English radiology reporting, indicating that the model has learned this convention during generation. Additionally, the preference for terms such as ‘solid lesion’ instead of ‘solid mass’, which are semantically equivalent yet constitute more commonly used clinical expressions, suggests that the model performs semantic reformulation with terminological flexibility. The conversion of long or segmented sentences from the original report into more fluent and structurally balanced forms further demonstrates that the language model performs syntactic optimization during English text generation. The more orderly expression of accompanying findings and the accurate contextual preservation of differential diagnostic cues such as “Lung CA?” indicate that the model not only rewrites the findings but also maintains the decision-support function of the report within a coherent linguistic structure.

To complement the automatic NLP metrics reported above, a structured radiologist evaluation of the model-generated reports was performed. The same board-certified thoracic radiologist who produced the dataset annotation rated the reports generated for all 77 test patients on a five-point Likert scale (1 = very poor, 5 = excellent) across five clinically meaningful dimensions: anatomical accuracy (correctness and completeness of anatomical references), pathological completeness (coverage of the tumor and accompanying parenchymal findings), terminology correctness (proper use of radiological terms), linguistic fluency (naturalness of clinical phrasing), and clinical usefulness (direct usability of the report in clinical decision-making). For each test patient, the original Turkish reference report was provided alongside the model-generated Turkish and English reports, and each language-specific output was rated independently. The results are summarized in Table 8.

Across the five evaluation dimensions, the model-generated reports achieve an overall mean rating of 4.21 ± 0.62 in the Turkish scenario and 3.92 ± 0.74 in the English scenario, indicating that the generated reports are judged to be clinically useful in the great majority of cases. The highest scores are observed in linguistic fluency (TR: 4.45, EN: 4.15), reflecting the strong language-modeling prior carried over from the pretrained GPT-2 checkpoints, while the most modest scores are recorded for clinical usefulness (TR: 3.94, EN: 3.68), the most stringent and subjective of the five dimensions, which captures the residual challenge of integrating subtle clinical reasoning into the generated text. The cross-language gap remains uniformly narrow (between 0.26 and 0.36 across all dimensions, with an overall difference of 0.29), and the largest single gap occurs in terminology correctness (Δ = 0.36), reaffirming that the differences between the two scenarios are concentrated in the translation-induced canonicalization of distinctive radiological terms rather than in any structural deficit of the model. Together with the BLEU, ROUGE, METEOR, and CIDEr scores reported in Table 7, these radiologist ratings provide convergent evidence that the proposed framework produces reports that are not only lexically aligned with the reference texts but also clinically meaningful in expert assessment.

#### 3.3.3. Computational Performance

To address the practical deployment requirements of the proposed framework, the inference cost was measured directly on the RTX 4090 workstation used for training. Each measurement reports a complete patient-level forward pass, comprising the encoding of five 224 × 224 axial slices through the ViT and LQAP modules and the autoregressive generation of a 64-token report through the GPT-2 decoder. The benchmark was averaged over 50 independent runs after a 10-iteration warmup phase. As summarized in Table 9, the model contains approximately 253.13 million trainable parameters and requires approximately 564.74 GFLOPs per patient.

The wall-clock inference time on a single RTX 4090 GPU is 343.5 ± 5.8 ms per patient (median 344.9 ms, P95 351.0 ms), corresponding to a throughput of 2.91 patients per second, with a peak GPU memory footprint of only 1.04 GB. These figures indicate that the framework can produce a complete diagnostic report and explainability map in less than half a second per patient, that the memory footprint remains compatible with mid-range clinical workstations, and that the system can, in principle, process more than 10,000 patients per hour on a single GPU, providing a level of throughput that aligns with the radiology demand of typical high-volume centers.

## 4. Discussion

This study demonstrates that an explainable, multi-slice transformer architecture can achieve clinically meaningful performance in both lung cancer classification and automatic radiology report generation tasks. The proposed framework reconstructs, within a transformer-based paradigm, the two-stage cognitive workflow of human radiologists: first identifying the lesion visually and then translating these findings into a structured textual report. The overall accuracies of 97.40% (Scenario 1, Turkish) and 94.81% (Scenario 2, English) on the independent 77-patient test set, together with the strong AUC values across all three classes, indicate that the model possesses robust diagnostic discrimination capability on histopathologically confirmed cases.

**Figure 9:**
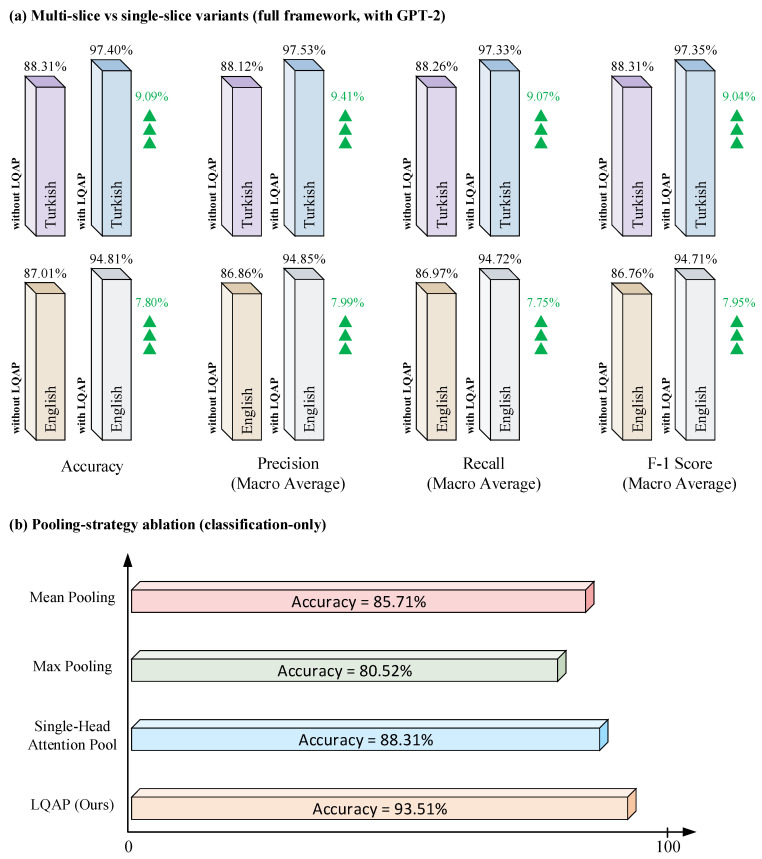
Two complementary ablations of the proposed framework: (**a**) Multi-slice vs. single-slice variants of the full ViT + LQAP + GPT-2 pipeline; only the input dimensionality (T = 1 vs. T = 5) changes between variants. Performance is reported on the same 77-patient test set across four metrics (Accuracy, macro Precision, macro Recall, macro F1) and both reporting scenarios (Turkish and English). Green upward arrows with adjacent percentages denote the absolute performance gain obtained by integrating LQAP. (**b**) Pooling-strategy ablation, classification-only (no GPT-2 decoder). Four cross-slice aggregation modules (Mean Pool, Max Pool, Single-Head Attention Pool, and the proposed LQAP) are compared under identical five-slice input conditions, the same ViT-Base/16 encoder, and the same training protocol on the same 77-patient test set.

The architectural value of the multi-slice design and of the LQAP module itself was assessed through two complementary ablations summarized in Figure 9. The first ablation (Figure 9a) keeps the full ViT + LQAP + GPT-2 pipeline intact and changes only the input dimensionality (T = 1 instead of T = 5), so that the resulting four-metric drop across both reporting scenarios isolates the contribution of multi-slice volumetric encoding from the rest of the framework. The second ablation (Figure 9b) holds the input fixed at five slices and replaces LQAP with three simpler aggregation schemes (mean pooling, max pooling, and single-head attention pooling) within the same ViT-Base/16 encoder and classification head. Because the simpler pools collapse the entire 985-token sequence into a single patient-level vector, they cannot directly feed the cross-attention sublayers of the GPT-2 decoder, which require a sequence of context tokens; this second ablation is therefore reported in a classification-only setting in order to isolate the pooling contribution. Within that classification-only setting, LQAP reaches 93.51% accuracy, exceeding the strongest parameter-free pool (Mean Pool, 85.71%) by 7.80 percentage points and the single-head attention pool (88.31%) by 5.20 percentage points. The remaining gap to the 97.40% headline accuracy of Section 3.3.1 reflects the additional contribution of the cross-attention pathway and the joint language-modeling objective, which together act as a domain-aware regularizer of the shared 768-dimensional latent space; the present ablation deliberately removes that pathway in order to expose the standalone effect of the pooling design.

**Figure 10:**
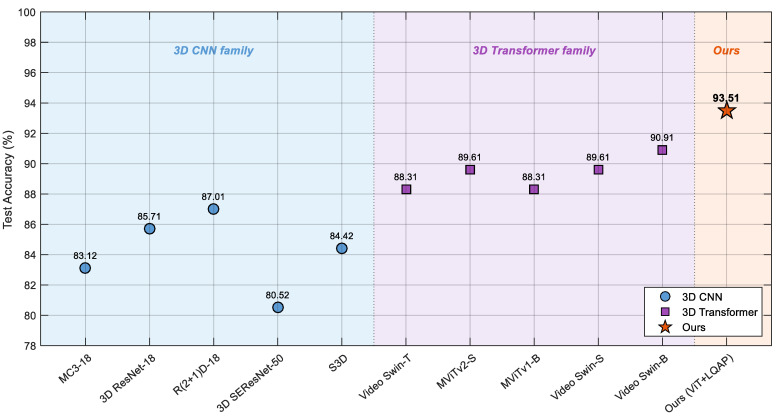
Visual side-by-side accuracy comparison of the proposed framework against ten representative 3D-CNN and 3D-transformer baselines on the LungCA 77-patient test set.

To benchmark the proposed framework against representative state-of-the-art volumetric models, ten additional 3D baselines were trained on the LungCA dataset under exactly the same input pipeline, train/test split, optimizer, scheduler, batch size, and number of epochs. Four 3D-CNN variants (MC3-18, 3D ResNet-18, R(2+1)D-18, and 3D SEResNet-50) operate directly on the native 5-slice patient input, whereas the remaining six baselines (S3D, Video Swin-T/S/B, MViTv1-B, MViTv2-S) architecturally require longer temporal windows and were therefore fed inputs upsampled to 16 frames via trilinear interpolation. All torchvision.models.video baselines were initialized from publicly available pretrained weights, the monai-based 3D SEResNet-50 was trained from scratch under identical hyperparameters, and only the final linear classification head was replaced in each case. To ensure a strictly head-to-head comparison, the proposed framework was evaluated in the same classification-only setting as the baselines, so that all eleven entries reflect the classification capacity of the visual encoder alone. The full set of results is summarized in Table 10 and visualized in Figure 10. The proposed framework reaches 93.51% accuracy on the 77-patient test set, exceeding the strongest 3D-CNN baseline (R(2+1)D-18, 87.01%) by 6.50 percentage points and the strongest 3D-transformer baseline (Video Swin-B, 90.91%) by 2.60 percentage points. A clear family-level separation is also visible: the 3D-transformer baselines (mean accuracy 89.35%) consistently outperform the 3D-CNN baselines (mean accuracy 84.16%) by approximately 5.2 percentage points, confirming the advantage of attention-based long-range modeling for multi-slice CT analysis. The remaining 2.60 pp accuracy gap between the strongest 3D-transformer and our framework, taken together with the pooling ablation discussed above, supports the architectural value of the latent-query aggregation strategy over what generic 3D-transformer designs achieve when adapted to thoracic CT classification.

By integrating both classification and report generation within a single architecture, the framework offers substantially enhanced clinical functionality compared with conventional CAD systems that produce only categorical predictions. The dual-task structure jointly optimizes classification and language-modeling losses, which both accelerates the diagnostic workflow and improves consistency in the generated reports. Two complementary forms of evidence support the clinical adequacy of these reports: the automatic NLP metrics in Table 7 confirm strong lexical and structural alignment with the reference reports, while the structured radiologist Likert evaluation summarized in Table 8 (overall mean ratings of 4.21 ± 0.62 in Turkish and 3.92 ± 0.74 in English across five clinically meaningful dimensions) confirms that the generated reports are judged clinically useful in the great majority of cases. The highest scores fall on linguistic fluency (TR: 4.45, EN: 4.15) and the most stringent on clinical usefulness (TR: 3.94, EN: 3.68), consistent with the residual difficulty of integrating subtle clinical reasoning into the generated text.

**Figure 11:**
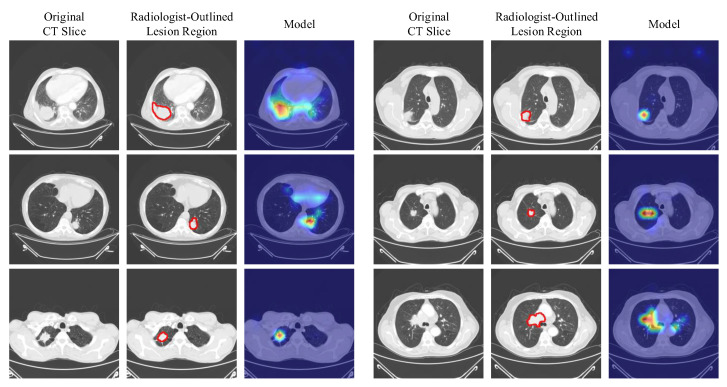
Comparison of radiologist-annotated regions with model-generated attention maps on external CT images. Red contours denote the lesion boundaries manually delineated by the radiologist.

The framework’s clinical applicability is further strengthened by its decision-confidence structure and by the spatial coherence of its visual explanations. The maximum-softmax confidence distributions in Figure 6 show that correctly classified patients cluster tightly within the 0.90–1.00 probability band, while misclassifications are confined to a narrow mid-probability region, indicating that the model does not produce overconfident outputs and reflects uncertainty in exactly the cases where lesion morphology is least discriminative. The Grad-CAM analyses in Figure 7 show that high-confidence predictions are supported by anatomically relevant regions and that the strongly focused attention patterns align with tumor mass and diagnostically important auxiliary indicators rather than with bony or clinically irrelevant tissues, in contrast to many published explainability studies. This spatial focus is also quantitatively verified in Table 6: against expert-annotated bounding regions on all 51 malignant test patients (255 annotated slices), the model achieves a mean IoU of 0.45 ± 0.14, a mean Dice of 0.59 ± 0.15, and a Pointing Game accuracy of 85.4%, with slightly higher localization scores for SCLC (IoU 0.48) than for NSCLC (IoU 0.42), consistent with the more centrally located morphology of small-cell lesions. Figure 11 extends this evidence outside the curated cohort: on CT images from an external source not included in the LungCA dataset, the model’s attention maps remain consistent with radiologist-annotated tumor regions, suggesting that the learned attention behavior transfers to data acquired under different conditions.

The same confidence pattern also speaks to inter-patient variability: the fact that misclassifications are confined to a narrow mid-probability region rather than appearing throughout the distribution indicates that the model’s confidence is governed by lesion discriminability rather than per-patient idiosyncrasies, and that the cross-slice aggregation effectively absorbs anatomical and acquisition-related variation across the 77-patient cohort. Consistently, no class shows systematically lower performance, with macro-averaged F1 scores of 97.35% (TR) and 94.70% (EN) across patients drawn from a heterogeneous clinical cohort. Several architectural choices further support generalization beyond this curated dataset. The cross-attention pathway between the LQAP context and the GPT-2 decoder grounds every generated token in the visual evidence rather than in patient-identifying surface features, reducing the risk of memorization-driven overfitting; the multi-task formulation with the classification loss (Equation (20), λ = 0.3) acts as an additional regularizer that biases the shared 768-dimensional latent space toward diagnostically meaningful structure; and the standardized lung-window preprocessing together with the ImageNet-pretrained ViT backbone insulates the framework from scanner-specific intensity profiles and provides broad low-level visual priors.

The performance gap between the Turkish and English scenarios deserves a more detailed examination, since it carries information about both the linguistic asymmetry of the dataset and the behavior of the cross-modal pipeline. Within the dataset, the Turkish reports serve as the reference, whereas the English versions are derived through professional medical translation of those original Turkish texts. Even with high-quality professional translation, the resulting English text inevitably introduces a small amount of lexical and structural drift relative to the source: idiomatic radiological phrasings characteristic of Turkish-language reporting are partially canonicalized into more uniform English expressions, and the agglutinative morphology of Turkish, which packs diagnostic modifiers tightly into single multi-morpheme tokens, is replaced by more periphrastic English constructions that distribute the same information across additional surface forms. This asymmetry, rather than any fundamental limitation of the model, explains the bulk of the observed gap.

The classification results show that this drift manifests most clearly at the boundary between the two malignant categories. In the Turkish scenario, all errors are confined to the SCLC class (one case predicted as Normal and one as NSCLC), and the SCLC-NSCLC boundary is otherwise crossed in only a single direction. In the English scenario, the same SCLC-Normal and SCLC-NSCLC errors are present, an additional SCLC case is also assigned to NSCLC, and a new NSCLC-to-SCLC confusion appears that has no counterpart in the Turkish scenario. The added errors are therefore concentrated almost entirely in the malignant-versus-malignant region of the decision space, which is precisely where the most lexically distinctive radiological cues (centrality of the mass, peribronchial extension, ground-glass components, spiculated margins) are most affected by translation-induced canonicalization. The confidence distributions in Figure 6 are consistent with this interpretation: the additional English-side errors cluster in the mid-probability band (approximately 0.50 to 0.93) rather than at high confidence, indicating that the model itself signals uncertainty in exactly those cases where translation has compressed the discriminative cues.

The report-generation metrics reveal a parallel pattern with an internally consistent structure. BLEU-1 is essentially identical across the two scenarios (0.512 versus 0.509), confirming that single-word reproduction is preserved by translation; BLEU-4 even shows a marginal English advantage (0.235 versus 0.233), which is plausibly explained by the more canonical phrase forms that professional translation tends to produce. The Turkish advantage becomes evident only at metrics that reward longer, semantically coherent matches: ROUGE-L (0.574 versus 0.514, +11.7%) measures the longest common subsequence and is therefore sensitive to narrative flow; METEOR (+7%) credits stem-level and synonym alignment, which is again reduced when surface forms shift through translation; and CIDEr (+11.5%) up-weights diagnostically distinctive expressions, which are precisely the terms most likely to be smoothed during translation. Taken together, the picture is coherent: the visual representation space learned by the model is itself language-agnostic, and short-range lexical reproduction transfers cleanly between scenarios, while longer-range diagnostic narrative coherence and rare clinical phrasings reflect the language in which the reference reports were originally written. The fact that the English outputs nonetheless retain strong structural and semantic accuracy confirms the adaptability of the proposed framework to multilingual clinical environments.

The end-to-end inference characteristics measured in Section 3.3.3 also bear directly on how the proposed framework could be integrated into existing clinical workflows. In a standard radiology environment, thoracic CT volumes are stored in the institutional Picture Archiving and Communication System (PACS) in DICOM format and indexed through the Radiology Information System (RIS). The framework can be deployed as a service that subscribes to new thoracic CT studies via DICOM C-STORE or DICOMweb (QIDO/WADO/STOW) interfaces, applies the same lung-window preprocessing and 5-slice landmark-based selection used during training, and returns its outputs in formats consumable by routine viewers and reporting tools. The classification result and the per-slice Grad-CAM saliency maps can be encoded as DICOM Secondary Capture or DICOM Segmentation objects so that they appear directly as overlays in standard PACS viewers, while the generated narrative report can be returned to the RIS as a draft note in HL7 v2 or HL7 FHIR DiagnosticReport format. The wall-clock inference time of 343.5 ± 5.8 ms per patient (Table 9) is well below the typical reading latency of a thoracic CT, so the AI-generated draft can be made available to the reporting radiologist effectively in real time, before the radiologist begins manual interpretation. From an information-security standpoint, on-premise deployment on a hospital-internal GPU server (which the modest 1.04 GB memory footprint comfortably permits) keeps all patient images within the institutional perimeter and avoids the regulatory complications associated with external cloud transfer.

The translation of the proposed framework into clinical use also raises ethical considerations that warrant explicit discussion across three dimensions. With respect to bias, although the architectural choices listed above are designed to minimize dataset-specific overfitting, residual demographic, equipment-related, and acquisition-protocol biases stemming from the single-institution, single-scanner origin of the dataset cannot be ruled out and should be assessed prospectively on multi-center cohorts before any deployment claim is made. With respect to data privacy, all imaging data used in this study were retrieved from the institutional PACS archive in DICOM format and irreversibly anonymized at the source: every header field containing patient-identifying information was removed prior to analysis, and no identifier traceable to an individual patient was retained at any stage of model training, validation, testing, or report storage; the study protocol was reviewed and approved by the Firat University Ethics Committee (protocol code 2024/13-38), the work was conducted in accordance with the ethical principles of the Declaration of Helsinki, and future deployments in distributed clinical settings would benefit from privacy-preserving techniques such as federated learning that allow multi-center training without transferring raw patient images outside their institutions of origin. Finally, with respect to clinical responsibility, the framework is intended as a decision-support tool that operates alongside qualified radiologists rather than as an autonomous diagnostic system: the model produces a classification, an attention map, and a draft radiology report, all of which require independent verification by a board-certified physician before being communicated to patients or used in treatment decisions, and final clinical responsibility for any diagnostic determination remains, in all settings, with the responsible radiologist and clinical team. Any prospective integration of the framework into routine practice will additionally require regulatory approval and the establishment of clear governance procedures for handling model-generated outputs that are inconsistent with subsequent expert review.

Several limitations of the present study should be acknowledged. First, the dataset originates from a single Turkish clinical center, with all CT volumes acquired on a single scanner model; while the architectural choices discussed above mitigate the most direct forms of dataset-specific overfitting, definitive cross-institutional and cross-scanner generalization cannot be claimed without prospective multi-center validation. Second, all class labels were verified by a single board-certified thoracic radiologist; although every malignant label was anchored to a histopathological gold standard, the absence of a second independent reader precludes the calculation of inter-rater agreement statistics, and future work will incorporate multi-reader annotation to enable formal reliability analysis. Third, the report-generation component is presently restricted to sentences pertaining to the lung parenchyma; future work aims to extend the system to produce comprehensive thoracic reports that also cover mediastinal, pleural, and cardiovascular structures. Fourth, the bilingual asymmetry analyzed earlier in this Discussion arises from translation-derived English reports; constructing a parallel cohort of independently authored English reference reports would allow disentangling translation-induced canonicalization from genuine model behavior.

## 5. Conclusions

This study demonstrates that an explainable, multi-slice transformer architecture can operate with clinically meaningful accuracy in the diagnosis of lung cancer using computer-assisted assessment. The proposed framework integrates, within a single unified structure, the processing of volumetric CT data, the execution of three-class diagnostic classification, and the automated generation of radiology reports from imaging inputs. Through this design, the framework addresses two persistent limitations in the literature: the reliance on single-slice models and the limited clinical utility inherent to purely categorical outputs. The high overall accuracies and the strong AUC values achieved across all three classes on the independent test set demonstrate that the model can consistently interpret volumetric lung morphology. In particular, LQAP mechanisms enable the model to capture anatomical continuity across sequential CT slices, producing a volumetric representation that surpasses the capabilities of traditional CNN-based approaches. As a result, the model is able to process lesion boundaries, surrounding parenchymal changes, and tumor-specific morphological patterns in a clinically coherent manner. The dual-task architecture, which jointly optimizes classification and linguistic generation, provides strong integration in translating image-based clinical content into structured text. The decoder component successfully generated clinically consistent, semantically aligned, and terminologically accurate reports in both Turkish and English; these outcomes were quantitatively validated through ROUGE-L, BLEU, METEOR, and CIDEr scores. The results further indicate that the visual representation space operates in a language-agnostic manner and that the model holds strong potential for enhancing standardization within multilingual clinical environments. Moreover, Grad-CAM attention maps showed that the model’s high-confidence decisions were associated with anatomically correct and pathologically meaningful regions. This study introduces one of the first comprehensive multi-slice transformer-based frameworks that unifies classification, report generation, and explainability within a single pipeline for thoracic CT analysis. By transforming volumetric imaging data into structured clinical narratives, the proposed approach has the potential to reduce workload in high-volume centers, increase reporting consistency, and support diagnostic workflows in settings with limited radiologist availability. While prospective multi-center validation, formal regulatory clearance, and structured real-world deployment testing under continued radiologist oversight remain necessary before routine clinical adoption, the present framework provides a concrete foundation for integrated multi-slice classification, radiology report generation, and visual explanation in thoracic imaging.

## Figures and Tables

**Table 1:** Selected state-of-the-art studies on lung cancer classification using CT imaging.

Study	Dataset	Number of Classes	Method	Performance	Explainability	Report Generation
Priya et al. [28]	LUNA-16	3 (Malignant, Benign and Normal)	CNN (SE-ResNeXt-50) Single Slice	Accuracy = 99.15% Precision = 99.51% Recall = 97.58% F-1 Score = 98.54%	Black-box	N/A
Shatnawi et al. [29]	Kaggle Chest CT	4 (Adenocarcinoma, Large cell carcinoma, Squamous cell carcinoma, Normal)	CNN (Custom 8-layer Model) Single Slice	Accuracy = 100% Precision = 99.20% Recall = 98.00% F-1 Score = 98.40%	Black-box	N/A
Mannepalli et al. [30]	Kaggle Chest CT	4 (Adenocarcinoma, Large cell carcinoma, Squamous cell carcinoma, Normal)	Transformer (gSC-DViT) Single Slice	Accuracy = 99.69% Precision = 99.60% Recall = 99.24% F-1 Score = 99.40%	Black-box	N/A
	IQ-OTH/NCCD	3 (Malignant, Benign and Normal)		Accuracy = 99.69% Precision = 99.60% Recall = 99.24% F-1 Score = 99.40%		
Gulsoy and Kablan [31]	IQ-OTH/NCCD	3 (Malignant, Benign and Normal)	Hybrid (FocalNeXt) Single Slice	Accuracy = 99.81% Precision = 99.78% Recall = 99.36% F1-Score = 99.56%	Black-box	N/A
Ozdemir et al. [32]	IQ-OTH/NCCD	3 (Malignant, Benign and Normal)	Hybrid (CNN + ViT) Single Slice	Accuracy = 99.54% Precision = 99.67% Recall = 99.60% F-1 Score = 99.12%	Grad-CAM	N/A
	Kaggle Chest CT	4 (Adenocarcinoma, Large cell carcinoma, Squamous cell carcinoma, Normal)		Accuracy = 98.41% Precision = 98.61% Recall = 98.35% F-1 Score = 98.48%		
Siam et al. [33]	LIDC-IDRI + IQ-OTH/NCCD + Kaggle	3 (Malignant, Benign and Normal)	CNN (FVCM-Net) Single Slice	Accuracy = 98.26% F1-Score = 97.37%	SHAP and HiResCAM	N/A
Raza et al. [34]	IQ-OTH/NCCD	3 (Malignant, Benign and Normal)	CNN (EfficientNetB1) Single Slice	Accuracy = 99.10% Precision = 99.22% Recall = 97.22% F1-Score = 98.16%	Grad-CAM	N/A
Hammad et al. [35]	Kaggle Chest CT	4 (Adenocarcinoma, Large cell carcinoma, Squamous cell carcinoma, Normal)	CNN (Custom Model) Single Slice	Accuracy = 93.06% Precision = 95.53% Recall = 93.09% F1-Score = 93.84%	Grad-CAM	N/A
Dhanya and Kumar [36]	IQ-OTH/NCCD	3 (Malignant, Benign and Normal)	Hybrid (MS-MambaNet) Single Slice	Accuracy = 96.80% Precision = 96.79% Recall = 96.80% F1-Score = 96.78%	Grad-CAM	N/A
	LIDC-IDRI	2 (Malignant and Benign)		Accuracy = 95.10% Precision = 95.30% Recall = 95.00% F1-Score = 95.15%		
This study	LungCA (Turkish Report)	3 (SCLC, NSCLC and Normal)	Transformer (ViT + LQAP + GPT) Multi Slice	Accuracy = 97.40% Precision = 97.53% Recall = 97.33% F1-Score = 97.35%	Grad-CAM	TR and EN
	LungCA (English Report)			Accuracy = 94.81% Precision = 94.85% Recall = 94.72% F1-Score = 94.71%		

**Table 2:** Tensor shapes and roles along the encoder-to-decoder pipeline.

Stage	Tensor Shape	Role
ViT encoder output (per slice)	(B, 197, 768)	Slice-level patch tokens (1 CLS + 196 patches)
Multi-slice concatenation	(B, 985, 768)	5 slices stacked along the token dimension
LQAP-Latent output	(B, 197, 768)	Patient-level visual context
LQAP-Patient output	(B, 768)	Single patient embedding for classification head
Decoder text input	(B, T, 768)	Sum of token and positional embeddings
Cross-attention queries (Q)	(B, T, 768)	Projected from text representation
Cross-attention keys/values (K, V)	(B, 197, 768)	Projected from LQAP-Latent context
Cross-attention output	(B, T, 768)	Visually conditioned text representation
LM head logits	(B, T, 50,257)	Next-token logits over GPT-2 vocabulary

**Table 3:** Hyperparameters of the proposed framework.

Components	Hyperparameter	Value
Encoder	Input Resolution	224 × 224
	Patch Size	16 × 16 pixels
	Number of Tokens	197
	Embedding Dimension	768
	Number of Transformer Layers	12
	Number of Attention Heads	12
	Positional Embedding	Learnable
	Attention Dropout	0.1
	MLP Dropout	0.1
	Residual Dropout	0.1
	Activation Function	GELU
	Pre-trained Backbone	vit_base_patch16_224
	Trainable Parameters	85.80 M
Decoder	Embedding Dimension	768
	Number of Decoder Layers	12
	Number of Attention Heads	12
	Max Token Length	1024
	Activation Function	GELU
	Attention Dropout	0.1
	MLP Dropout	0.1
	Embedding Dropout	0.1
	Cross-Attention Mechanism	Enabled
	Positional Encoding	Learnable embeddings
	Tokenizer	TR and EN
	Trainable Parameters	152.81 M
LQAP (Latent)	Input Dimension	(B, 985, 768)
	Output Dimension	(B, 197, 768)
	Number of Learnable Latent Queries	197
	Attention Heads	12
	MLP Expansion Ratio	4×
	Dropout	0.1
	Normalization	LayerNorm
	Latent Query Initialization	Xavier uniform
	Trainable Parameters	7.24 M
LQAP (Patient)	Input Dimension	(B, 197, 768)
	Output Dimension	(B, 768)
	Number of Learnable Class Queries	1
	Attention Heads	12
	MLP Expansion Ratio	4×
	Dropout	0.1
	Normalization	LayerNorm
	Latent Query Initialization	Xavier uniform
	Trainable Parameters	7.09 M
Total Trainable Parameters		253.13 M

**Table 4:** Evaluation metrics and formulas.

Category	Metric	Mathematical Definition
Classification	Accuracy	$\frac{TP+TN}{TP+TN+FP+FN}$
	Precision	$\frac{TP}{TP+FP}$
	Recall	$\frac{TP}{TP+FN}$
	F1-Score	$\frac{2\cdot Precision\cdot Recall}{Precision+Recall}$
Report Generation	BLEU-n	$BP\cdot \exp\left(\sum_{n=1}^{N}w_{n}\ln\left(p_{n}\right)\right)$
	ROUGE-L	$\frac{\left(1+\beta^{2}\right)\cdot LCS}{\left|Ref\right|+\left|Can\right|}$
	METEOR	$F_{mean}\cdot \left(1-p\right)$
	CIDEr	$\frac{1}{M}\sum_{m=1}^{M}CIDEr_{m}$

**Table 5:** The performance values obtained across both scenarios.

Class	Metric	Scenario-1 (TR)	Scenario-2 (EN)
Normal	Precision	96.30%	96.30%
	Recall	100%	100%
	Specificity	98.04%	98.04%
	F-1 Score	98.11%	98.11%
SCLC	Precision	100%	95.65%
	Recall	92.00%	88.00%
	Specificity	100%	98.08%
	F-1 Score	95.83%	91.66%
NSCLC	Precision	96.30%	92.59%
	Recall	100%	96.15%
	Specificity	98.04%	96.08%
	F-1 Score	98.11%	94.34%
Overall	Accuracy	97.40%	94.81%
	Macro Precision	97.53%	94.85%
	Macro Recall	97.33%	94.72%
	Macro Specificity	98.69%	97.40%
	Macro F-1 Score	97.35%	94.70%

**Table 6:** Quantitative validation of Grad-CAM saliency maps against radiologist-annotated tumor regions across all 51 malignant test patients.

Class	n (Slices)	IoU (Mean ± SD)	Dice (Mean ± SD)	Pointing Game Accuracy
SCLC	125	0.48 ± 0.12	0.62 ± 0.14	88.8%
NSCLC	130	0.42 ± 0.15	0.57 ± 0.16	82.2%
Overall	225	0.45 ± 0.14	0.59 ± 0.15	85.4%

**Table 7:** Report generation performance for both scenarios.

Model	BLEU-1	BLEU-2	BLEU-3	BLEU-4	ROUGE-L	METEOR	CIDEr
Scenario-1 (TR)	0.512	0.413	0.316	0.233	0.574	0.272	0.493
Scenario-2 (EN)	0.509	0.389	0.295	0.235	0.514	0.254	0.442

**Table 8:** Radiologist Likert ratings (1–5 scale) of model-generated reports across 77 test patients.

Dimension	Turkish (TR)	English (EN)
Anatomical Accuracy	4.28 ± 0.55	4.02 ± 0.68
Pathological Completeness	4.05 ± 0.72	3.78 ± 0.81
Terminology Correctness	4.32 ± 0.48	3.96 ± 0.65
Linguistic Fluency	4.45 ± 0.51	4.15 ± 0.62
Clinical Usefulness	3.94 ± 0.83	3.68 ± 0.88
Overall (all dimensions)	4.21 ± 0.62	3.92 ± 0.73

**Table 9:** Computational performance of the proposed framework, measured on a single NVIDIA GeForce RTX 4090 GPU.

Metric	Value
Visual encoder (ViT + LQAP) FLOPs	90.67 G
Decoder FLOPs	474.07 G
Total FLOPs per patient	564.74 G
Inference time, mean ± SD	343.5 ± 5.8 ms
Inference time, median	344.9 ms
Inference time, P95	351.0 ms
Throughput	2.91 patients/s
Peak GPU memory	1.04 GB

**Table 10:** Head-to-head comparison of the proposed framework with ten representative 3D-CNN and 3D-transformer baselines on the LungCA test set.

Model	Family	Input T	Accuracy (%)	Macro F1 (%)
MC3-18	CNN	5 (native)	83.12	83.05
3D ResNet-18	CNN	5 (native)	85.71	85.60
R(2+1)D-18	CNN	5 (native)	87.01	86.93
3D SEResNet-50	CNN	5 (native)	80.52	80.20
S3D	CNN	16 (int.)	84.42	84.30
Video Swin-T	Transformer	16 (int.)	88.31	88.25
MViTv2-S	Transformer	16 (int.)	89.61	89.54
MViTv1-B	Transformer	16 (int.)	88.31	88.19
Video Swin-S	Transformer	16 (int.)	89.61	89.59
Video Swin-B	Transformer	16 (int.)	90.91	90.89
Ours (ViT + LQAP)	Transformer	5 (native)	93.51	93.49

## Data Availability

The data presented in this study are available upon request from the corresponding author.

## Abbreviations

The following abbreviations are used in this manuscript:

CNNs	Convolutional neural networks
CT	Computed tomography
K	Key
KDE	Kernel density estimation
LN	Layer normalization
LQAP	Learnable Query Attention Pooling
MHSA	Multi-head self-attention
MLP	Multilayer perceptron
NSCLC	Non-small-cell lung cancer
Q	Query
SCLC	Small-cell lung cancer
V	Value
ViT	Vision Transformer
